# The role of epigenetic modifications in drug resistance and treatment of breast cancer

**DOI:** 10.1186/s11658-022-00344-6

**Published:** 2022-06-28

**Authors:** Mohsen Karami Fath, Ali Azargoonjahromi, Arash Kiani, Fateme Jalalifar, Parisa Osati, Mahsa Akbari Oryani, Fateh Shakeri, Farhad Nasirzadeh, Behman Khalesi, Mohsen Nabi-Afjadi, Hamidreza Zalpoor, Maysam Mard-Soltani, Zahra Payandeh

**Affiliations:** 1 Department of Cellular and Molecular Biology, Faculty of Biological Sciences, Kharazmi University, Tehran, Iran; 2 Shiraz University of Medical Sciences, Shiraz, Iran; 3 Student Research Committee, Yasuj University of Medical Sciences, Yasuj, Iran; 4 School of Medicine, Jiroft University of Medical Sciences, Jiroft, Iran; 5 Chemical Engineering Department, Fouman Faculty of Engineering, College of Engineering, University of Tehran, Fouman, Iran; 6 Department of Pathology, Faculty of Medicine, Mashhad University of Medical Sciences, Mashhad, Iran; 7 Department of Research and Production of Poultry Viral Vaccine, Razi Vaccine and Serum Research Institute, Agricultural Research Education and Extension Organization, Karaj, Iran; 8 Department of Biochemistry, Faculty of Biological Science, Tarbiat Modares University, Tehran, Iran; 9 Shiraz Neuroscience Research Center, Shiraz University of Medical Sciences, Shiraz, Iran; 10 Network of Immunity in Infection, Malignancy and Autoimmunity (NIIMA), Universal Scientific Education and Research Network (USERN), Tehran, Iran; 11 Department of Clinical Biochemistry, Faculty of Medical Sciences, Dezful University of Medical Sciences, Dezful, Iran; 12 Department Medical Biochemistry and Biophysics, Division Medical Inflammation Research, Karolinska Institute, Stockholm, Sweden

**Keywords:** Breast cancer, Epigenetic modifications, microRNAs, Treatment, Chemoresistance

## Abstract

**Background:**

Breast cancer is defined as a biological and molecular heterogeneous disorder that originates from breast cells. Genetic predisposition is the most important factor giving rise to this malignancy. The most notable mutations in breast cancer occur in the *BRCA1* and *BRCA2* genes. Owing to disease heterogeneity, lack of therapeutic target, anti-cancer drug resistance, residual disease, and recurrence, researchers are faced with challenges in developing strategies to treat patients with breast cancer.

**Results:**

It has recently been reported that epigenetic processes such as DNA methylation and histone modification, as well as microRNAs (miRNAs), have potently contributed to the pathophysiology, diagnosis, and treatment of breast cancer. These observations have persuaded researchers to move their therapeutic approaches beyond the genetic framework toward the epigenetic concept.

**Conclusion:**

Herein we discuss the molecular and epigenetic mechanisms underlying breast cancer progression and resistance as well as various aspects of epigenetic-based therapies as monotherapy and combined with immunotherapy.

## Background

Breast cancer has become the most diagnosed cancer and one of the leading causes of cancer-related deaths worldwide [1]. It accounted for 2.3 million cases and 685,000 deaths in 2020 [2]. Estrogen is responsible for a wide range of functions, from reproductive system development to metabolic and mitotic regulation. Four subtypes of estrogen during menopause, reproductive years, fetal life, and pregnancy, are estrone, 17β-estradiol, estetrol, and estriol, respectively. So, the predominant form of estrogen varies depending on the life stage. Estrogen exerts its effects via estrogen receptors (ERs). Three types of ER, that is, ERα, ERβ, and G-protein-coupled estrogen receptor 1 (GPER1), have already been discovered. Estrogen binding to its ER could trigger various genomic and nongenomic signaling cascades. ER overactivation contributes to tumor proliferation and malignancy [3]. On the basis of ER, progesterone receptor (PR), and HER2 presentation, breast cancers are usually divided into three types, namely hormone receptor positive (ER^+^, PR^+^, HER2^+^), HER2 positive (ER^−^, PR^−^, HER2^+^), and triple negative. There are also six subtypes regarding the molecular classification of breast cancer cells: luminal A, luminal B, HER2, normal-like, basal-like, and claudin-low [4]. Approximately 70% of cases are hormone responsive with a good prognosis, while triple-negative cases account for a small percentage with a poor prognosis and progressive nature.

Having improved our knowledge of cancer biology notwithstanding, treatment of breast cancer has remained a challenging issue [5–7]. Of interest, the molecular anomaly of breast cancer cells cannot be explained via the genetic background as a result of the frequent sporadicity associated with the cancer [8]. However, epigenetic processes such as DNA methylation and histone modification, as well as microRNAs (miRNAs), have a noticeable impact on different dimensions of breast cancer [9–13]. Moreover, epigenetic details can be deemed as a way to gain knowledge about the molecular basis of breast cancer pathogenesis, improve therapeutic strategies, and develop novel therapeutic tools against breast cancer [14, 15].

In the past two decades, new therapies such as immunotherapy and targeted therapies have emerged as a result of the discovery of new players and pathways. However, a significant portion of patients remain resistant to therapy or experience recurrence, suggesting an incomplete understanding of cancer metabolism [14]. Given these circumstances, a thorough review of molecular and epigenetic mechanisms underlying breast cancer progression, resistance, and various aspects of existing epigenetic therapies in combination with immunotherapy methods would bring about new insights for biologists and clinicians. In this regard, we conducted a study to cover recent advances in breast cancer biology and anti-breast cancer therapies, with a focus on epigenetic approaches.

## Major signaling pathways in breast cancer

All cells, irrespective of type, need to be controlled by various signaling pathways to survive. Cells can communicate with one another or even with their environment via these signaling pathways [16, 17]. Many of these signaling pathways interfere within different cells and conditions, such as hijacking by cancer cells. As cancer cells undergo genetic and epigenetic alterations, hyperactivation of some signaling pathways can be exerted by aberrant mutations in tumor cells. Thus, the mutations are under the control of signaling pathways to curb the proliferation, survival, and migration of tumor cells [18]. Incidentally, inhibition of some pathways, such as the hexosamine biosynthetic pathway, was also found to cause breast cancer growth [19]. Herein, some of the most important signaling pathways affecting cellular functions of breast cancer will be discussed in detail.

### Estrogen receptor (ER) signaling

Estrogen receptors (ERs) are divided into membrane and nuclear receptors. The nuclear estrogen receptors include ERα and ERβ, which are capable of either inhibiting or animating the expression of target genes. Also, both ERα and ERβ play a role in transcription, the main feature of ERs. Generally, ERs can trigger interactions between ER dimers and estrogen response elements (EREs) of target cells, leading to enhanced transcription regulation. Many other co-inhibitors and co-animators affect the transcription process. As the level of ERα expression is remarkably higher than that of ERβ in breast cancer [20], it appears that ERα has a significant role in the pathogenesis (cross-talk) of breast cancer. A high percentage of patients (nearly 75%) were shown to have positive expression of ERα [21]. Notably, inhibition of ERα leads to activation of BRCA1 and, subsequently, suppression of tumor cell proliferation [20, 22].

ERα enhances the growth rate of breast cancer cells via interaction with cyclin D1 [23, 24]. Cyclin D1 has the ability to regulate the cell cycle transition from G1 to S phase in several tumor cells, as it acts as an enhancer of cyclin-dependent kinases (CDKs) 4 and 6 [25]. Therefore, the interaction between ERα and cyclin D1 has been considered the most important mechanism of ERα to enhance the growth rate of breast cancer cells [22]. ERα is categorized into different isoforms that, depending on their structure, could have varying impacts on ERα signaling [26–28]. The ERα36 isoform is reported to promote metastasis and exacerbate the disease among patients with breast cancer. ERα tamoxifen is an antagonist of ERα used to treat breast cancer. However, it has an adverse impact on ERα36 via upregulation of the *ALDH1A1* gene. Thus, tamoxifen in fact enhances the progression of the cancer instead of suppressing it [29].

As breast cancer cells proliferate, the expression of ERβ tends to decrease [30]. According to many studies carried out both in vivo and in vitro, ERβ suppresses the progression of breast cancer [31, 32]. For instance, in the p53-knockout mouse model, Igor Bado et al. found that ERβ exerts its tumor-suppressing function via interaction with p53 [33].

### HER2 signaling

**Fig. 1:**
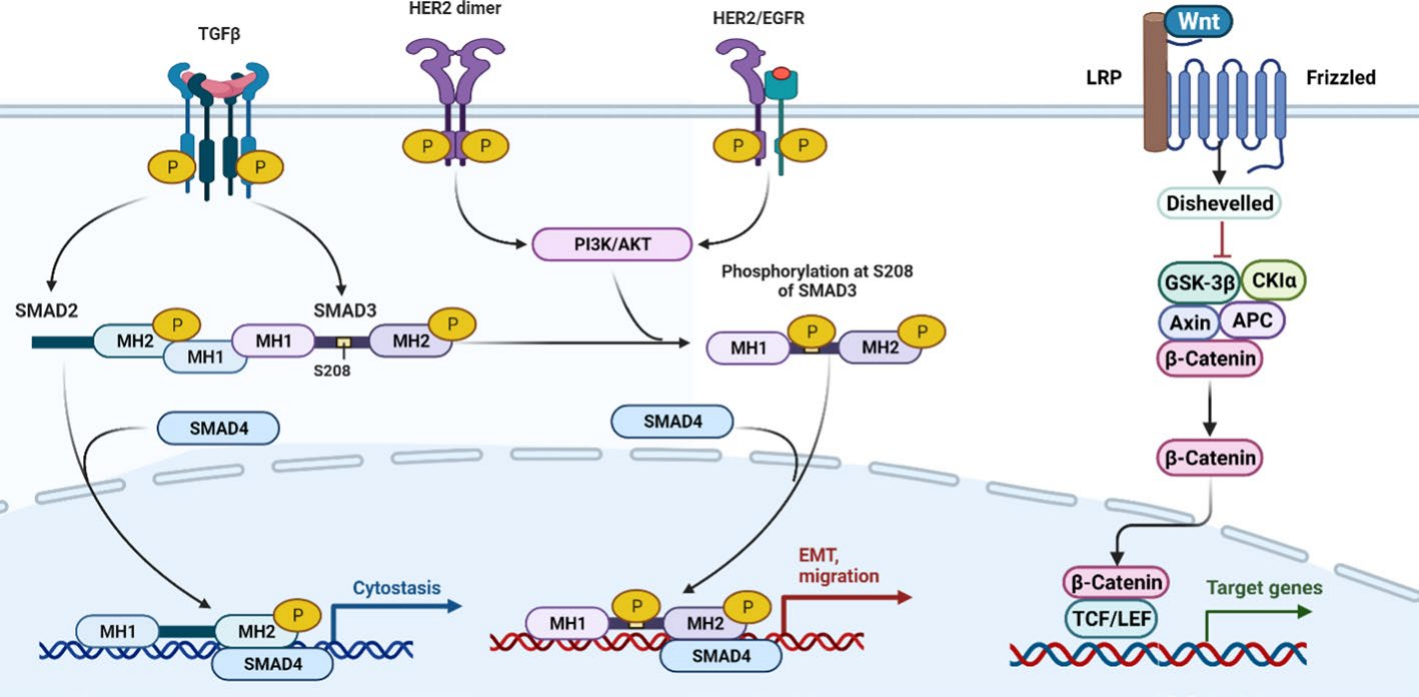
HER2 and Wnt/β-catenin signaling are two essential pathways with essential roles in the progression of breast cancer cells. HER2 signaling exerts its function via dimerization and thereby activation of various other pathways, such as PI3K/AKT, leading to EMT migration and, thus, cancer progression. The function of TGFβ can culminate in cytostasis and prevent cancer progression. Even though it can enhance phosphorylation as S208 of SMAD3, it amplifies the PI3K/AKT signaling pathway to promote breast cancer. In addition, the Wnt/β-catenin pathway can act as a breast cancer promoter and exacerbate the disease by entering β-catenin into the nucleus and affecting TCF/LEF

HER2 is a human epidermal growth factor receptor as well as a tyrosine kinase receptor [26, 28]. Activation of various signaling pathways such as mitogen-activated protein kinase (MAPK) and phosphatidylinositol-3-kinase (PI3K) can be triggered by phosphorylation of tyrosine residues in the intracellular domain of HER2. Such pathways are tightly linked to tumorigenesis [34–36]. Thus, HER2 overexpression and signaling are strongly correlated with cancer cell proliferation [37]. The precancerous effect of HER2 signaling in breast cancer is engendered by its association with inflammation and amplification of cancer stem-like cells (CSCs) [38]. Expression of HER2 gives rise to metastasis due to its promotion and prompting of migration in primary tumor cells and mammary stem cells, respectively, through signals originating from progesterone and progesterone-induced paracrine mechanisms [31] (Fig. 1).

Thus, targeting HER2 with monoclonal antibodies or tyrosine kinase inhibitors (TKIs) shows promising results in the treatment of patients with breast cancer. These treatments are more prominent among patients suffering from HER2^+^ neoplasms [39, 40]. In total, 15–20% of patients showed positive outcomes in response to such treatments [41].

### Wnt/β-catenin signaling

Wnt proteins have considerable roles in cellular processes, including embryonic progression, cell polarity generation, and retention of adult tissue homeostasis. Wnt proteins, palmitoylated by porcupine, can bind to both frizzled and low-density lipoprotein receptor-related protein 5 and 6 (LRP5/6) coreceptors. This binding initiates Wnt/β-catenin signaling. Interaction of Wnt and receptor leads to Axin and Disheveled proteins being recruited to the cell membrane and inhibits glycogen synthase kinase (GSK)-3β protein. β-Catenin can trigger GSK-3β to negatively regulate the Wnt pathway [34–36, 42, 43]. Notably, GSK-3β inhibition causes accumulation of β-catenin in the cytoplasm and paves the way for its translocation into the nucleus. Therefore, GSK-3β can act as a cotranscriptional enhancer, T-cell factor/lymphoid enhancing factor (TCF/LEF), and oncogene promoter [44, 45].

Wnt1 and Wnt3 are not shown to be expressed in adult mammary glands, whereas other members of the Wnt family—Wnt-2, 4, 5a, 5b, 6, and 7—can be expressed at different stages of mammary development [46–48]. Expression of Wnt-2, 5a, and 7b has been observed in virgin mouse mammary glands. These molecules are downregulated during pregnancy, thereby inducing the expression of Wnt-4, 5b, and 6 during pregnancy [49].

In breast cancer, an autocrine mechanism can activate Wnt signaling. A high level of stabilized β-catenin is prevalent among breast cancer cases (roughly 50%) [39, 40]. Wnt inhibitors such as frizzled-related protein 1 (FRP1) and dickkopf 1 (DKK1) are shown to be associated with metastatic processes and poor prognosis in breast cancer cases [50, 51]. The Wnt/β-catenin pathway has been shown to be noticeably activated in basal-like breast tumors, and its nuclear localization is significantly correlated with poor prognosis. Activated β-catenin promotes triple-negative breast cancer (TNBC), the most malignant subtype of breast cancer [11]. Moreover, it plays a crucial role in the in vivo induction of HER2 mammary tumors [22]. Ayyanan et al. [52] found that stem cell self-renewal can be increased by expressing Wnt1 in human mammary epithelial cells. This means higher resistance against apoptosis and senescence [53]. To confirm the critical role of the Wnt pathway in the progression of breast cancer cells, various investigations have been carried out. These investigations commonly revealed that inhibition of Wnt1 can change the phenotypes to CD44^+^CD24^−^ALDH1^−^ and diminish both tumor formation and cellular migration [54]. Moreover, GSK3/β-catenin can be suppressed by the inhibition of protein kinase D1 (PRKD1). This event subsequently reduces the cellular function of breast cancer [55]. Therefore, Wnt signaling plays a pivotal role in the preservation of mammary stem cell properties (Fig. 1).

## Epigenetic structure in breast cancer

### DNA methylation

DNA methylation has been defined as a process by which methyl groups are reversibly added to the fifth carbon position of the cytosine from *S*-adenosyl methionine (SAM). This process is accomplished by DNA methyltransferases (DNMTs) [44] such as DNMT1, DNMT3A, and DNMT3B. Some differences have been detected among these DNMTs. For instance, DNMT1 (known as maintenance DNA methyltransferase) solely adds methyl groups to the hemimethylated DNA generated during DNA replication, whereas both DNMT3A and DNMT3B establish new DNA methylation patterns during embryogenesis (known as de novo methylation patterns) [45].

The correlation between DNA methylation patterns and the pathogenesis of breast cancer was revealed by whole-genome methods. These investigations have led to the identification of 345 methylated genes in 40 different breast cancer lines [56]. Holm et al. attempted to find a link between the variation of methylome pattern and breast cancer heterogeneity. They found that nearly 18,700 genes have differential methylation [57–60], linked with the target CpG, a DNA site where a cytosine nucleotide is followed by a guanine nucleotide in the linear sequence of bases along its 5′ → 3′ direction [48]. Unlike other cancers, in breast cancer, hypomethylation has been detected in gene bodies [46]. It is also noteworthy that, unlike in the promoter, where CpG methylation is negatively correlated with gene expression, CpG methylation in the gene body can culminate in transcriptional activation [46, 47].

Noteworthily, some genes involved in cell differentiation, DNA binding, homeobox proteins, and transcription signaling are influenced by DNA methylation. DNA methylation also affects some processes involved in the growth of neoplastic cells, including chromatin remodeling, transcriptional control, DNA repair, cell-cycle control, apoptosis, and metabolism [61]. Furthermore, changes in DNA methylation lead to the altering of cell adhesion, tissue invasion, and metastasis pathway genes [62]. According to a study carried out by the Mathot team, DNA methylation influences the tumor microenvironment, leading to changes in tumorigenesis–metastasis properties [63].

### Histone modification

**Fig. 2:**
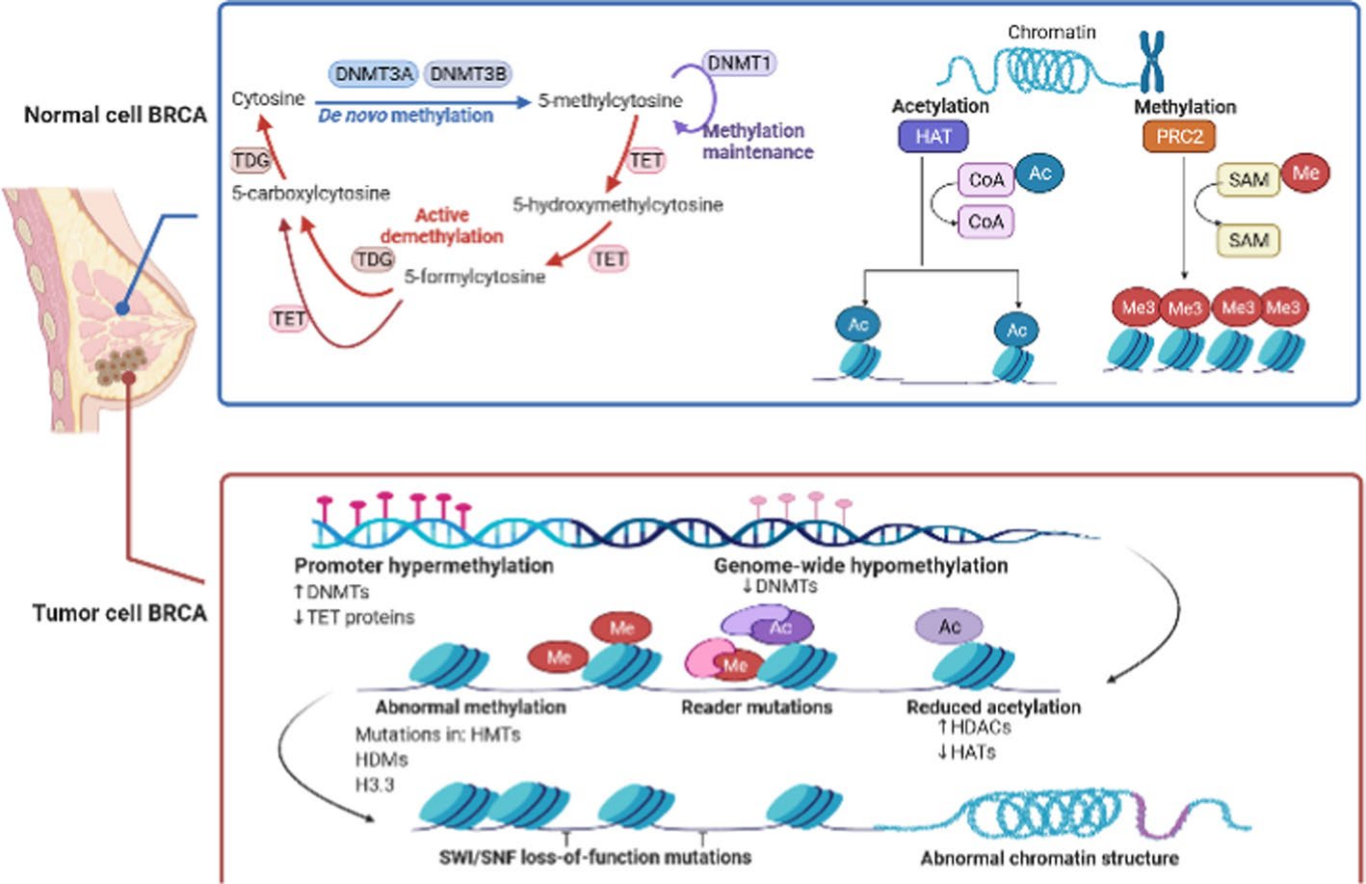
Epigenetic changes in routine and tumor cells in breast cancer. Epigenetic modifications such as DNA methylation and histone modification alter DNA accessibility and chromatin structure, thereby regulating patterns of gene expression. In normal cells, DNA methyltransferases (DNMTs) add a methyl group at position 5 of the pyrimidine ring of cytosine, which is known as DNA methylation. The Ten Eleven Translocation (TET) family of proteins catalyze the subsequent oxidations of 5-methylcytosine (5mC) to 5-hydroxymethylcytosine (5hmC), 5-formylcytosine (5fC), and 5-carboxylcytosine (5caC). TET proteins therefore provide an active pathway for DNA demethylation and consequently have relevance for regulation of gene expression. TET proteins can also mediate active demethylation via excision of 5fC and 5caC by thymine DNA glycosylase (TDG). Additionally, histone acetyltransferases (HATs) acetylate conserved lysine amino acids on histone proteins by transferring an acetyl group from acetyl-CoA to form ε-*N*-acetyllysine. Also, the methylation of histone H3 at lysine 27 (H3K27me1/2/3) is catalyzed by polycomb repressive complex 2 (PRC2)—which is a chromatin-modifying enzyme. Moreover, both gene transcriptional repression and normal organismal development can be maintained by this complex. Nonetheless, cancer cells have a different epigenetic profile. Decreased methylation was seen in the epigenetic profile of cancer cells. This decreased methylation can affect the activity of large numbers of genes. Since methylation has been linked with decreased gene activity, the implication of hypomethylation is to escalate the activity of the affected genes. In this condition, if genes involved are in cell growth, this event can result in cell division and, thus, cancer progression. In addition, increased activity of histone deacetylases (HDACs) has been found in different types of cancer cells such as breast cancer, and it is marked by a loss of histone acetyl markers. Histone methyltransferases (HMTs) are enzymes that add methyl groups to histones, while histone demethylases (HDMs) have the inverse function. In breast cancer cells, HMTs may be altered so that they place methyl groups at the wrong spot, thereby silencing tumor suppressor genes. All these events as well as mutation of SWI/SNF chromatin remodeling complex subunits can lead to abnormal chromatin structure, contributing to cancer development

Histone modifications such as acetylation, deacetylation, and methylation have been subjected to thorough investigation in the case of breast cancer. Histone acetylation occurs when an acetyl group is covalently added to the amino group of lysine from an acetyl-CoA molecule [64]. Histone deacetylation is defined as the removal acetyl groups from lysine residues in the NH2-terminal tails of core histones, which leads to a more closed chromatin structure and repression of gene expression [65], whereas histone methylation is defined as the addition of a methyl group to lysine and arginine residues in the histone tail [53]. Along with findings indicating a strong linkage between histone modification and tumor prognosis, it has also been shown that histone acetylation and methylation markers vary between normal cells and breast cancer cells [66]. Moreover, a marker to recognize breast cancer subtypes has been identified. H3K4me3, H3K27me3, and H3K27me3 depletion can be found in Luminal A, HER2, and basal subtypes of breast cancer, while H3K27me3 enrichment was solely observed in Luminal A subtype [67, 68]. This issue has led to the idea of clustering among breast cancer subtypes. For instance, basal subtypes can be divided into three different clusters, according to genome-wide K27me3/K9me3 ± K14ac level [69–71] (Fig. 2).

Histone modification profile of frozen tissue was shown to have remarkable differences from the primary cell lines. Moreover, histone modification of patient-derived breast cancer tissue, primary breast cancer cells, and established breast cancer cell lines has been profiled into three H3 lysine acetylations, 14 H3 lysine methylations, and 14 combinatorial lysine modifications. Depending on the alteration in the histone modification, cell line types may differ from one another. For instance, K36me1 or K9me3/K14Ac deposition was observed among breast cancer cell lines, while primary cells tended to be deprived of the K14Ac marker [72]. Recently, numerous studies were carried out to ascribe histone modification to tumorigenesis, cancer progression, and metastasis of breast cancer. According to the attained findings, approximately 650 genes were detected to push normal cells toward neoplastic transformation. H3K9 posttranslational modification affects neoplastic transformation in cases of breast cancer that use a transformation model to express the Large T antigen, TERT, and RAS (V12). Furthermore, tumorigenic transformation can be marked by a significant decline in the H3K9me2/me3 in tandem with H3K9ac accumulation [73]. The impact of imbalanced histone modification (such as H3K9ac/H3K27me3 dysregulation) on tumorigenesis, tumor cell progression, breast cancer signaling, and metastasis has been investigated. A correlation between H3K9ac imbalance and genes related to cell proliferation, apoptosis regulation, cell–cell signaling, cell migration, and metabolic processes has also been revealed. Moreover, cell cycle-associated genes are enriched with H3K27me3, while its decline is directly linked to drug resistance and resistant-like cells [74, 75].

Histone deacetylases (HDACs) have been classified, on the basis of their homology with yeast proteins, into four classes. HDACs can regulate the expression and activity of many fold proteins involved in both cancer initiation and progression. HDAC1 was shown to be overexpressed in breast cancer [76]. Of note, the overexpression of HDAC1, HDAC6, or HDAC8, in breast cancer cell lines, was shown to be associated with increasing cell invasion and matrix metallopeptidase 9, which is a protein-coding gene known as MMP9 [77]. In addition, HDAC7 ascribes to cell growth via cooperation with ERα to repress Reprimo—a cell cycle inhibitor and tumor suppressor gene [78].

### MicroRNAs (miRNAs)

Numerous studies indicate that miRNAs are associated with breast cancer pathogenesis irrespective of its stage. It should be highlighted that the impact of miRNAs is considered a double-edged sword, that is, miRNAs tend to have oncogenic and tumor-suppressive effects [61]. After analyzing the expression profiles of 309 miRNAs, 133 miRNAs were shown to affect the normal and cancerous tissues of different breast cancer subtypes. miRNAs can also be used to inform breast cancer stages. This information could lead to the prediction of tumor relapse risks and, hence, the survival rate of patients with breast cancer (Table 1) [57–60].

**Table 1:** miRNAs and their function in breast cancer [57–60]

Function	miRNAs
Tumor stage and/or metastasis	miRNA-10b, miRNA-34a, miRNA-373, miRNA-21, and miRNA-155
Prediction tumor relapse	miRNA-18b, miRNA-103, miRNA-107, and miRNA-652
Systemic treatment success	miRNA-342-3p and miRNA-187-3p
Progression-free survival	miRNA-93, miRNA-195, miRNA-20b, miRNA-342-3p, and miRNA-187-3p

In addition, miRNAs are capable of controlling estrogen receptor (ER) expression. This means that miRNAs have pivotal roles in both normal breast development and breast tumor formation. Downregulation of ER expression by miRNA-142-3p, miRNA-335-5p, miRNA-21, and miRNA-192-5p is essential for direct miRNA function in ER expression. Likewise, some miRNAs such as miRNA-148a reduce DNMT1 expression, which leads to upregulation of ER expression in MCF-7 cells [61]. In contrast to the aforementioned miRNAs, others, such as miRNA-27a, act as oncogenic factors to induce overexpression of ER and exacerbate cancer progression [58].

## Role of epigenetic and miRNAs in breast cancer development and pathogenesis

Cell proliferation could be either induced or inhibited by miRNAs. Unlike miR-143, miR-455, miR-424, miR-26a-5p, and miR-543, which inhibit cell proliferation, miR-1207-5p expression is the consequence of increasing the level of a long noncoding RNA PVT1. It can explicitly be shown that increased and decreased cell proliferation can promote and inhibit tumor cell progression, respectively (Table 2) [46].

**Table 2:** Role of miRNAs in pathogenesis by either increasing or decreasing cell proliferation [46]

Main function	miRNAs	Function of miRNAs
Increasing cell proliferation	miR-1207-5p	Regulating STAT2 expression Inactivating cell-cycle-dependent kinase inhibitors including CDKN1A and CDKN1B
	miR-492	Upregulating cyclin D1 and c-MYC Repressing the expression of SOX7, deemed as a transcription factor
	miR-135b	Negatively regulating LATS2 tumor suppressor kinase and the Hippo pathway
Decreasing cell proliferation	miR-143	Suppressing the expression of extracellular signal-regulated kinase ERK5 Suppression of mitogen-activated protein kinase MAP3K7 and cyclin D1
	miR-455	A double-knockdown impact of Cdc2-related protein kinase CDK14 Cyclin D1 expression Promoting expression of tumor suppressor p21
	miR-424	Binding to its selective target, cyclin-dependent kinase *CDK1* in G2-M cell phase The expression of the Yes-associated protein YAP by both the Hippo pathway and p-ERK1/2 of the ERK pathway
	miR-543	Direct regulating of ERK/MAPK pathway
	miR-26a-5p	Reducing the expression of cell cycle regulators cyclin D1, CDK4, and CDK6 Increasing the expression of tumor suppressor proteins such as p21, p27, and p53

Correlation with EMT and regulation of genes, which are related to cell motility and invasion, is imperative for metastasis of tumor cells. miRNA can establish this correlation. For instance, the miR-200c/141 cluster has metastatic potential in breast cancer cells. These miRNAs have the ability to regulate the expression of SerpinB2, which is linked to TNBC rather than the Luminal subtype. Since the elevation of different transcription factors such as C-Jun, c-Fos, and FosB mRNAs could cause the overexpression of miR-200c/141, they could increase the metastatic potential of the tumor. Given these facts, miR-200c/141 cluster and SerpinB2 can be considered prognostic indicators of TNBC cancer [47]. miRNAs could also influence the pathogenesis of tumors as a result of FOXP3 and KAT2B functions as coordinators. As an example, higher levels of miR-200 family, miR-200c, and miR-141 prompted by FOXP3 and KAT2B were seen in patients in the metastatic phase [61].

On the other hand, patients with metastasis were found to have downregulated levels of some miRNAs (such as miR-195) compared with patients suffering from localized breast cancer. Some studies have pointed out that miRNAs, which are correlated with metastatic genes, can promote tumorigenicity. miR-331, which is strongly correlated with genes relevant to metastasis such as *HER2*, *HOTAIR*, *E2F1*, *DOHH*, and *PHLPP*, is the epitome of such miRNAs promoting tumorigenicity [62].

Energy metabolism has been affirmed as the other mechanism facilitating metastasis in breast cancer. A high level of secreted miR-122 was ascribed to glucose metabolism in the premetastatic niche, leading to a significant escalation in the availability of nutrients in this niche. Therefore, miR-122 is deemed as a promoter of metastatic processes in breast cancer. It was also pointed out that miR-122 reduces glucose consumption among nontumor cells by downregulating pyruvate kinase (PKM) and citrate synthase (CS). These processes could provide a source of energy for tumor cells and hence the progression of breast cancer cells. In addition to the aforementioned, the Wnt/β-catenin signaling pathways are considered to promote or inhibit pathogenicity, depending on the sort of miRNAs affecting this pathway. For instance, miR-374a can suppress negative regulators of Wnt/β-catenin signaling pathway such as WIF1, PTEN, and WNT5A, which could activate the Wnt/β-catenin cascades. That could be the reason behind the elevation of miR-374a in metastatic breast cancer [79]. Conversely, miR-148 targets WNT-1 and decreases the expression of the other crucial components in the Wnt/β-catenin pathways such as β-catenin, MMP-7, and TCF-4 in breast cancer cells. Thus, miR-148 inhibits the migration and invasion of breast cancer cells [80]. Moreover, various miRNAs are involved in suppressing metastasis and invasion among breast cancer cells. Of note, miRNAs, such as miR-497, miR-421, miR-193a, miR-211-5p, miR-335, miR-133a, and miR-124, can prevent the tumor cells from metastasizing by suppressing the expression of SMAD7, MTA1, WT1, SETBP1, EphA4, LASP1, and STAT3 [66, 81–85].

The other function of miRNAs reported is the inhibition of immune responses and tumor immune escape, thus promoting tumor invasion. According to recent studies, miR-497 in tandem with miR-195 can target the 3′-UTR of the cluster of differentiation CD274 in TNBC cells. Therefore, miR-497 contributes to immune escape and decreases the immune response. Overexpression of miR-204-5p regulates genes correlated with immune pathways such as TNF and cytokine signaling. Upregulation of miR-240-5p could also alter the tumor immune microenvironment and remarkably reduce the number of immune cells. Nonetheless, the overexpression of miR-240-5p has been found to increase the number of CD4^+^ T cells, CD8^+^ T cells, and regulatory T cells [67]. Refraining from the apoptotic response is considered a vital feature to be obtained by tumor cells. This feature could be acquired by different mechanisms, including reducing tumor suppressor p53, dysregulating caspase activity, upregulating pro-survival regulators, downregulating pro-apoptotic factors, and deactivating death ligands [68]. Notably, miRNAs are divided into two categories: anti-apoptotic (miR-519a-3p and miR-191-5p) and apoptotic (miR-148a, miR-101, kallistatin, miR-204 miRNAs) [46]. miR-519a-3p and miR-191-5p are categorized as anti-apoptotic miRNAs, while miR-148a, miR-101, miR-204, and kallistatin act as apoptotic molecules [69–71, 86]. The functions of these miRNAs are exerted by various mechanisms, which will be discussed in the following sections.

It has already been shown that some miRNAs can inhibit tumor cells from undergoing apoptosis. miR-519a-3p enables the tumor cells to protect themselves from apoptosis induced by TNF-related apoptosis-inducing ligand (TRAIL) and Fas ligand. The other mechanism of miR-519a-3p to protect the tumor cells from apoptosis is the escape from natural killer (NK) cells. It reduces levels of MICA and ULBP2, displayed on the cell surface by NK cells [72]. miR-191-5p has also been shown to decrease both apoptosis and caspase-3/-7 activity. Apparently, the greater the downregulation of SOX4 by miR-191-5p, the lower the expression of P53 in breast cancer cells. This finding suggests that miRNAs could be targeted to treat breast cancer cells. For instance, a drug called doxorubicin (acting as anti-miR-191-5p) is used in cases of breast cancer with increased P53 expression to promote apoptosis [73]. Wang et al. indicated that miR-204 can induce apoptosis by targeting JAK2. This miRNA is also negatively correlated with p-STAT3, ending with the downstream anti-apoptotic proteins BCL-2 [75]. Noteworthily, miR-148a can reduce BCL-2 expression, thus promoting apoptosis [74]. Guan et al. pointed out that miR-101 can negatively target EYA1 expression via components of the Notch signaling pathway, such as jagged1, Hes1, and Hey1, which were significantly decreased as a result of miR-101 overexpression [57]. miR-101 can also suppress SOX2, resulting in inhibiting breast cancer growth and migration [60]. An endogenous protein, known as kallistatin, is capable of reducing the viability in breast cancer cells and augmenting the caspase-3 activity, which causes increased apoptosis. Kallistatin exerts its function through the augmentation of autophagy markers such as LC3B, Atg5, and beclin-1. Additionally, the expression of oncogenic miR-21 can be inhibited by kallistatin via suppressing the miR-21–Akt pathway. Also, it decreases the expression of anti-apoptotic BCL-2 and enhances the pro-apoptotic BAX. Kallistatin can decline the expression of oncogenic miR-203. It manages this process via PKC–ERK activation. Conversely, it is noteworthy that kallistatin triggers the expression of tumorigenic suppressors such as miR-34a and p53. These changes could lead to contrasting impacts on breast-cancer-mediated deaths [59, 87].

Telomeres consist of TTAGGG tandem repeats, which are located at the end of each chromosome, and their length affects apoptosis. Telomeres are also influenced by some miRNAs with critical roles in apoptosis [58]. It should be noted that the telomerase reverse transcriptase protein (hTERT) and the telomerase RNA template (ERC) are deemed crucial regulators of telomerase activities [48]. As previously reported, the lower expression of miR-512-5p/296-5p and the higher expression of hTERT in breast cancer cells culminates in poor clinical outcomes among patients with breast cancer [46]. The other miRNA that affects the telomere is miR-155; high expression of miR-155 results in telomere fragility by inhibition of TRF1 [88].

Hypoxia and angiogenesis are imperative for the progression of breast cancer cells. These processes can be triggered by miRNAs via their double-edged sword effects, that is, some miRNAs promote angiogenesis, while others inversely prevent it (Table 3).

**Table 3:** Impact of miRNA on angiogenesis under conditions of hypoxia

	miRNAs	Function of miRNAs	References
Increasing angiogenesis under hypoxia condition	miR-210-3p miR-191 miR-24	Linking to HIF sites by HIF-1α and HIF-2α chromatin immunoprecipitation (ChIP)-sequence analysis Stimulation of TGF-β-signaling pathways Increasing the level of genes pertaining to TGFβ-signaling pathways, namely TGFβ2, SMAD3, BMP4, JUN, FOS, PTGS2, CTGF, and VEGFA Increasing formation of mammospheres Escalating the expression of Nanog and Oct-3/4 stemness genes Decreasing the expression of pro-apoptotic BimL Increasing the levels of two HIF-1α direct targets, Snail and VEGFA	[47, 89, 90]
Decreasing angiogenesis under hypoxia condition	miR-140-5p miR-29b miR-497	Inhibiting VEGFA expression Decreasing the expression of proteins such as CD31, Ki-67, and MMP-9 Targeting AKT3 protein Promoting VEGF and c-MYC arrest Upregulating VEGF and HIF-1α	[91–93]

Changes in the expression of miRNAs are another factor controlling the progress of breast cancer cells. Most interestingly, miR-205-5p can target genes related to breast cancer invasiveness, such as *SOCS3*, *PTPRN2*, and *MMP3*; it also affects genes relevant to EMT regulatory functions such as TGF-β1. Thus, miR-205-5p can act as a pro-tumorigenesis factor [94]. miRNAs are also involved in tumor suppressor genes. For example, miR-498, miR-1297, and miR-103b trigger PTEN, which leads to breast tumorigenesis [95, 96].

Commodore miRNAs are a set of five miRNAs pertaining to the regulation of a large gene network involved in breast cancer. They consist of miR-190b, miR-let-7i, miR-292-b, miR-511, and miR-141. miR-190b is involved in the regulation of genes related to dynein assembly, vitamin metabolism, and proliferation of mammary gland epithelial cells. miR-let-7i modulates genes related to adaptive immune response and leukocyte cell–cell adhesion. miR-292-b regulates the genes related to melanocyte transport, angiogenesis, and epithelial cell migration. miR-511 regulates genes related to cytokine production. miR-141 controls genes associated with motility, migration, and extracellular matrix organization. These features are detected merely in breast cancer cells. Since no such miRNAs are found among healthy breast tissue, they can be used as novel biomarkers to monitor the trend of breast cancer progression, or even target therapeutic methods [97].

## Epigenetic impacts upon drug resistance in breast cancer

The US Food and Drug Administration (FDA) has already approved nine epidrugs to treat breast cancer. However, one-third of patients with early-stage ER^+^ breast cancer may experience treatment resistance. Notably, the efficacy of these medicines to defeat endocrine therapy resistance has been thus far a subject of intense debate [98]. This treatment resistance could be caused by enhanced preexisting niche tumor cells or dynamic reprogramming mediated by epigenetic alterations [99]. Endocrine therapy resistance is classified into intrinsic or acquired resistance [100]. Various players could contribute to this process, including genetic alteration, changes to mitogenic signaling pathways, alteration of genes encoding epigenetic factors, hypoxic conditions, and so forth. Herein, some mechanisms pertaining to resistance will be discussed in detail.

### Alterations of ESR1 and genes involving in estrogen-mediated signaling

Tumor cells are dependent on ERα to grow and survive, which is targeted by endocrine therapy. Ligand-independent reactivation of ERα has been deemed a primary mechanism of resistance [101]. Mutations in ESR1 (encoding ERα) can mediate ERα activation. It is worth noting that most ERα mutations are situated in two adjacent amino acids in the LBD. These mutations include the tyrosine at position 537 mutated to either asparagine, cysteine, or serine (ERαY537N/C/S) and aspartic acid at position 538 mutated to glycine (ERαD538G) [102]. Although ESR1 mutations can only be found in 1% of all patients, it is prevalent in metastases after endocrine therapy (accounting for about 20–40%). It also has a direct link to poor response to AI and tamoxifen [103]. The other genetic alteration is served by ESR1 gene fusion, which is enriched in ER^+^ breast cancer cases and deemed a novel driver of resistance. Owing to depriving ESR1 fusion proteins of the LBD, tumor cells undergoing such alterations have been shown to be insensitive to endocrine therapy [104]. Genetic alteration in CYP19A1 (gene encoding aromatases) increases the enzymatic activity of E2-independent ERα that binds to target cells after AI treatment. ERα can also be activated by overexpression of aromatases during the epigenetic reprogramming process [105].

FOXA1 (activated by gene amplification) has been considered as the genome-wide promoter of reprogramming among breast cancer cases undergoing endocrine resistance [106]. FOXA1 chromatin can be distributed from active promoters to de novo promoters holding AP-1, while resistance is acquired, in terms of tamoxifen resistance models. However, some inhibitors are able to overcome endocrine resistance relating to overexpressed FOXA1 by targeting FOXA1 downstream and HAF-2α [107].

### Cell-cycle alterations in endocrine-resistant breast cancer

Cell-cycle alterations play a crucial role in drug resistance. Moreover, the link between high levels of CDK4 and endocrine resistance within breast cancer cells has been identified [106]. The loss of tumor suppressors such as RB and FAT1 characterizes the resistance to CDK4/6 inhibition. Approximately 10–20 percent of these cases have remained insensitive, and a noticeable percentage [65–75] of them become resistant to treatment after 12–36 months of therapy. It is noteworthy that primary (2%), and metastatic (6%) breast cancer cells experience the loss of function of FAT1 as a receptor of the Hippo pathway. This event may result in enhanced CDK6 expression due to the YAP and ZAT recruitment into CDK6 enhancer and progression of G1/S [108–110]. In addition, hyperactivation of RTK–RAS signaling and aberrant activation of CCNE1-CDK2 as a CDK4/6 downstream effector results in the resistance and reduction of response to palbociclib [109]. Perhaps it is an amenable elucidation for the combination of CDK4/6 with immunotherapy or with endocrine therapy to be an appealing strategy in the treatment of patients with breast cancer. PI3K/AKT plays a crucial role in cell growth, proliferation, survival, and metabolism. This pathway is considered the other pathway responsible for treatment resistance in breast cancer cells. Furthermore, like PTEN loss, activated AKT is correlated with tamoxifen resistance [111].

### Alteration of ERα coregulators

It has been demonstrated that NF1 (neurofibromin) can drive endocrine therapy resistance via the merged impact of a loss of its GTPase activity and transcriptional corepressor roles of ERα. Since NF1 and NCoR1 can be recruited by tamoxifen, their level is linked to endocrine therapy agents to respond. Incidentally, a high level of both NF1 and NCoR1 is rampant among patients with metastatic ER^+^ breast cancer [112, 113].

### Epigenetic factors that contribute to endocrine-resistant breast cancer

ARID1A may mediate endocrine resistance. This property is due to the fact that ARID1A declines to access chromatin and bind TFs, which control the liminal cell fate. It also decreases the ERα and FOXA1 that bind to chromatin [114]. These observations result in a prevailing notion that therapeutic strategies should target the mutant ARID1A. Moreover, the reduced function of ARID1A and activated signaling of PI3K/AKT have been shown in endocrine resistance among patients with breast cancer. That is why EZH2 in ARID1A mutant should be targeted as a powerful therapeutic strategy in breast cancer treatment [103].

**Fig. 3:**
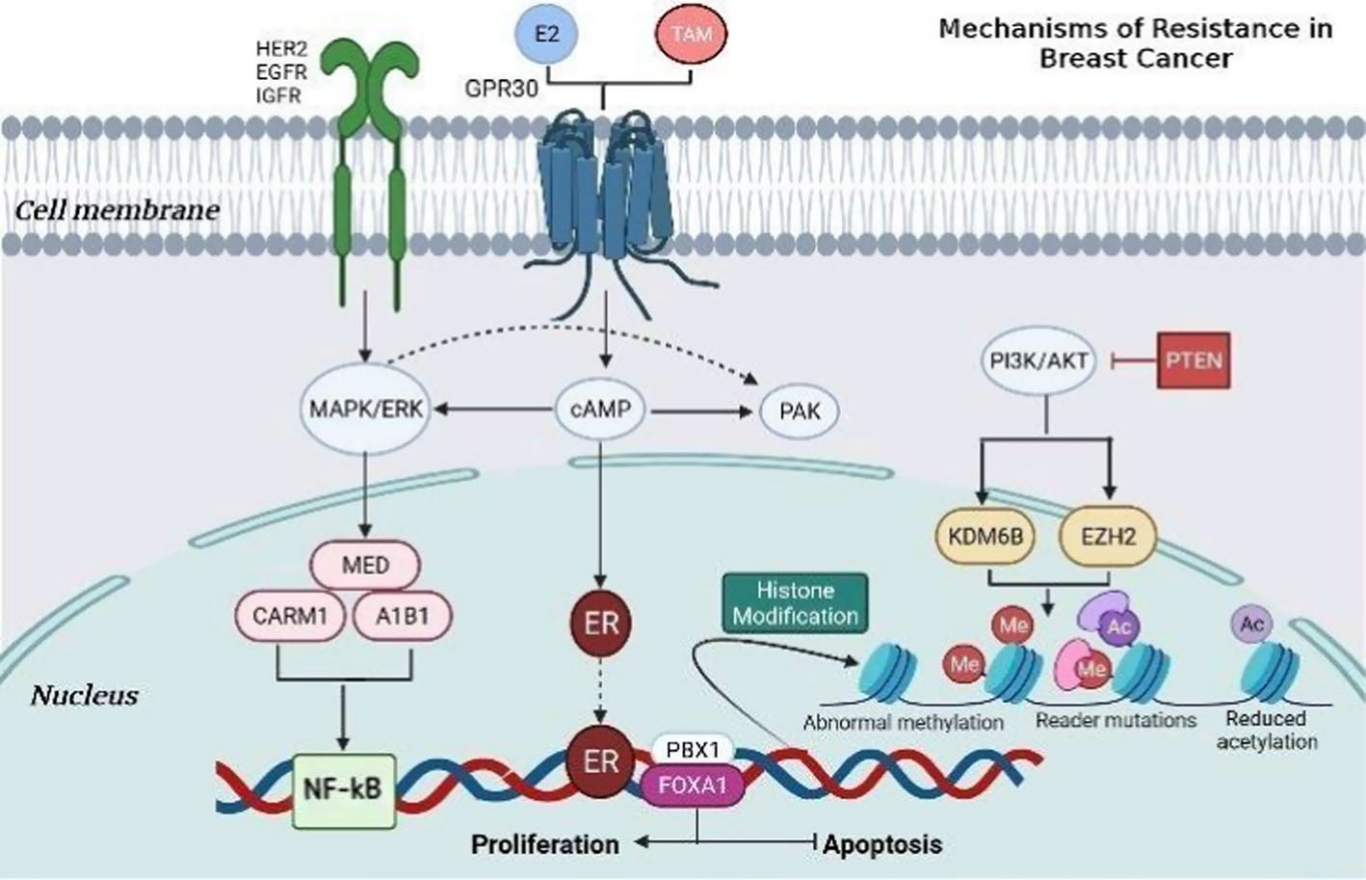
mechanism of resistance to endocrine therapy with tamoxifen (TAM) in breast cancer. Increasing the expression and activity of tyrosine kinase receptor family proteins, such as HER2, EGFR, IGFR, and GPR30, gives rise to alterations in translation signals and, thus, acquired resistance to TAM. These events lead to aberrant activation of cAMP/PKA, MAPK/ERK, and PI3K/AKT signaling pathways. Activation of these kinase pathways results in phosphorylation of ER and its co-activators such as A1B1, MED1, or CARM1, thereby activating proliferation and inhibiting apoptosis. Also, in the condition of deregulating ER, HER2-mediated signaling can be increased, and transcription factors such as Sp1, AP-1, and NF-κB can be activated, hence promoting oncogene transcription. Likewise, factors such as FOXA1 and PBX1 can recruit ER to specific genomic sites. In addition, mutations in the tumor suppressor protein have been seen to escalate phosphorylation of PI3K/AKT in ER^+^ tumors, resulting in therapeutic resistance. Incidentally, KDM6B and EZH2, which modify histones and cause aberrant protein phosphorylation, can lead to resistance to endocrine therapy from the reactivation of genes

DNA methylation and histone acetylation are deemed the most significant alterations to trigger cancer resistance. For instance, hypermethylation of ESR1 culminated in reduced ERα expression among approximately 20% of the patients administrated with tamoxifen [115]. Furthermore, HATs catalyze the acetylation of histones, transcription factor (TF)s, and heat shock proteins, leading to resistance in patients administered tamoxifen [116] (Fig. 3).

## Management of breast cancer by various therapy approaches

If breast cancer cases are categorized into early stages and metastatic groups, treatment of breast cancer can be managed by surgical resection, neoadjuvant/adjuvant chemotherapy, radiotherapy, endocrine therapy, and anti-HER2 therapy among early stages, and systemic therapy including endocrine therapy, target therapy, and cytotoxic chemotherapy within metastatic breast cancers [117]. Immunotherapy has been deemed a breakthrough in the treatment of breast cancer. Various methods of monotherapy in the treatment of patients with breast cancer are explained in the following sections.

### Targeting DNA methylation

Endogenous retrovirus (ERV) expression is triggered by inhibition of DNA methylation and is relevant to immune checkpoint blockade (ICB) response. Hence, clinical strategies could be developed to cure breast cancer by amalgamating DNMTi with ICB [118]. DNMTis can increase tumor antigenicity via various mechanisms such as re-expression of MHC molecules, tumor antigens, and changing cytokine production [119]. Accordingly, DNMTis induce the re-expression of endogenous ERα and PRs, and are highly efficient in inhibition of epithelial-to-mesenchymal transition and metastasis in TNBC cells [120]. Suppression of class 1 genes in breast cancer can be done by DNA methylation. However, DNMTi can reverse MHC1 gene promoter methylation and upregulate gene expression in response to interferon. Hence, using DNMTi could be an appealing strategy for breast cancer treatment [121]. Gaudecitabine (SGI-110) is a novel generation of DNMTi, which resists degradation by enzyme and cytidine deaminase. These properties provide a prolonged in vivo exposure and augment the efficacy [118–122]. Taken together, DNA methylation inhibitors such as azacitidine and decitabine have a remarkable impact on the performance of T cells and improve the revitalization of T cells. This is why the efficacy of the combination of azacitidine and decitabine, respectively, with durvalumab and pembrolizumab, should be examined to treat cases of breast cancer [118].

### Targeting histone de-acetylation

It was found that histone deacetylases (HDACis) increase the expression of MCH molecules on tumor cells, leading to the presentation of tumor-specific antigens [119]. Vorinostat, romidepsin, panobinostat, and belinostat are among the HDACis approved by the FDA [123]. There are few investigations to assess the effect of HDAC inhibitors in combination with immunotherapy regarding the treatment of breast cancer. However, it was pointed out that, in accordance with prior studies, HDACs could affect T-cell homeostasis, including the activation of regulatory T cells. FOXP3, for instance, suppresses IL-2 by interacting with HDAC3. Enhancing the effector T cells can be achieved by upregulation of RUNX3 as a result of HDAC1 and HDAC2 loss [124].

The activity of NK cells can be increased by upregulation of Natural Killer Group 2D (NKG2D) ligands as a result of consuming HDACis. However, the use of HDACis is associated with side effects such as lymphopenia. HDAC inhibitors are classified into four types of hydroxamate [e.g., suberoylanilide hydroxamic acid (SAHA)]. Owing to their toxicity, hydroxamates are considered as a part of combination therapy in breast cancer treatment. Furthermore, trichostatin A (TSA) is regarded as an antifungal antibiotic that represses HDAC activity and traps the cell cycle in G1 and G2 phases. It also induces cell differentiation, thus having a potent impact on the inhibition of tumor growth [118].

### Targeting histone demethylation and methylation

LSD1 (known as KDMA1) controls H3K4 and H3K9 demethylation. LSD1 inhibitors reprogram tumor-associated macrophages into M1-like ones owing to the methylation on H3K4 and H3K9. Stimulators of interferon gene (STING) correlated with intratumor CD8^+^ T cells by KDM5B and KDM5C are epigenetically silenced and, thus, immune latent [125]. Even though combination therapy with immunotherapy and histone demethylases has not yet been carried out, LSD1 could be coupled with ICB in the case of breast cancer [118]. Inhibition of EZH2 (an oncogenic histone methyltransferase) could increase the myeloid-derived suppressor cells (MDSCs) in the tumor microenvironment, thereby inhibiting the antitumor immunity. Nonetheless, there has been no investigation to evaluate this strategy in breast cancer [126].

### Targeting bromodomain in breast cancer

Bromodomain and extraterminal (BET) inhibitors effectively reduced NF-κB activity, cytokine generation, and PD-L1 expression in numerous studies. However, they increase the activity of NK cells [127].

Notably, reduced maturation of endotoxin-driven dendritic cells and generation of macrophage cytokine have been reported as the consequences of using BET inhibitors. This could be construed as antiinflammatory effects for these inhibitors [128]. BET inhibitors differentiate effector T cells into the effector memory phenotype via suppression of BATF. In TNBC, BET inhibitors can suppress both PD-1 and PD-L1 to prevent tumor-mediated T-cell exhaustion. Thus, a combination of immunotherapy and bromodomain inhibition may be beneficial. Moreover, the amalgamation of BET inhibitors with an anti-CTLA-4 agent was suggested as a practical approach, according to a mathematical model [118].

### Endocrine therapy

Estrogen receptor modulators (tamoxifen and fulvestrant) and aromatase inhibitors (anastrozole, letrozole, and exemestane) are two main types of hormone receptor drug employed in breast cancer treatment [129]. Depending on the menopausal status of the patients, the choice of an endocrine agent can be changed. For instance, owing to the ceased ovarian functions, estrogen is produced by fat and muscle cells in postmenopausal women, which leads to the use of aromatase inhibitors such as progestins and antiestrogens [130]. One of the drawbacks of this therapeutic approach is resistance to the treatment. For instance, upregulation of the PI3K/AKT/mTOR pathway and overexpression of cyclin D1 may occur, despite overcoming resistance by combining everolimus (as an mTOR inhibitor) with exemestane [131]. Combining therapies could improve progression-free survival (PFS), such as combining alpelisib with fulvestrant and CDK4/6 inhibitor with fulvestrant or aromatase inhibitors. Such treatment methods are well established in the cases of hormone receptor (HR)-positive breast cancer. It is important to understand that administration of endocrine therapy in advanced postmenopausal breast cancer can have side effects such as upper gastrointestinal distress, thromboembolic risk, fluid retention, stress incontinence, and withdrawal bleeding [107].

### Anti-HER2 therapy

Monoclonal antibodies such as trastuzumab, and pertuzumab, which inhibit HER1–HER2 dimerization, are among the anti-HER2 therapeutics and can cause an ADCC reaction. Ertumaxomab affects the HER2 on the tumor and T cells, which redirects the T cells, macrophages, dendritic cells, and natural killer cells to the sites of tumor metastasis [118]. Tyrosine kinase inhibitors (TKIs) such as lapatinib and neratinib are other types of anti-HER2 therapeutics. They can be used in early-stage breast cancer to treat HER-positive breast cancer. Neratinib is beneficial for the treatment of metastatic breast cancer. The antibody–drug conjugate (ADC) ado-trastuzumab emtansine (T-DM1) is considered to treat patients suffering from breast cancer, specifically in phase 2 [109].

### Cytotoxic chemotherapy

Adjuvant breast cancer chemotherapy results in a remarkable increase in molecular markers of cellular senescence, namely CDKN2a expression in PBTLs. Additionally, serologic levels of senescence-associated cytokines are enhanced by chemotherapy in the case of breast cancer. The impact of chemotherapy on ${P16}^{INK4a}$ expression has been confirmed in an independent, cross-sectional cohort of long-term breast cancer [108].

## miRNAs roles in the treatment of breast cancer

Since the impact of miRNAs on BCSCs is a double-edged sword, it has been the subject of intense debate. To clarify by an example, some miRNAs, including miR-221, miR-222, miR-140, miR-21, and miR-22, have carcinogenic effects, while the inverse is true for other miRNAs considered immensely potent to suppress tumor cells, namely miR-200 family, miR-128, miR-99a, miR-29b, Let-7 miRNA, miR-600, miR-34, and miR-30. miR-10b is considered as a biomarker to diagnose breast cancer in its metastatic stage. Moreover, the levels of miR-34a and miR-155 are increased in metastatic breast cancer cases. According to a small cohort study, miR-210 could be deemed a biomarker in lymph node metastasis [110]. miR-17 and miR-155 have been identified to be beneficial biomarkers to differentiate metastatic from nonmetastatic cases. Incidentally, miR-155 can inhibit CTLA-4 expression on T lymphocytes, which in turn could lead to the promotion of anticancer immune responses [111]. miR-200c could regulate the expression of BMI1, which is involved in the self-renewal of BCSCs. This miRNA is deemed a tumor suppressor and has been considered a potential therapeutic in the case of breast cancer [112].

miR-128 inhibits breast cancer progression by lowering the expression of BMI1 and ABCC5. Deletion of phosphatase expression and tensin homolog on chromosome 10 (PTEN) protein and inhibition of AKT phosphorylation can be suppressed by miR-221/222. Therefore, this miRNA is able to enhance the tumor-forming capability in breast cancer [113]. miR-30a is another miRNA that inhibits the growth of BCSCs by regulation of gene expressions of AVEN protein. The Notch pathway is essential for the regulation of self-renewal and apoptosis in BCSCs. The miR-200 family can suppress the Notch signaling pathway by targeting its components such as JAG1 and co-activators including Maml2 and Maml3 [114].

On the other hand, miR-9 and miR-34c have the ability to overexpress Notch signaling. Therefore, they can trigger a noticeable decline in the metastatic behavior of TNBC. Owing to miR-30e high expression, the self-renewal ability of BCSCs may be inhibited by suppressing the expression of Ubc9 [115, 116].

In contrast to what has been mentioned, miRNAs have pivotal roles in chemotherapy resistance. For instance, if miR-128 is downregulated, cancer cells may become resistant to chemotherapy. However, some miRNAs such as miR-200 and Let-7 are effective in improving sensitivity to chemotherapy. Thus, these miRNAs could be employed as predictors of drug resistance [117].

Turning to miRNAs roles in immunotherapy, it is of note that the antitumor efficacy of inhibiting CTLA-4 and PD-1 receptors and their corresponding ligand, PD-L1, has been reduced for a couple of reasons. First, solely one type of patient could benefit from using this strategy. Secondly, toxicity is rampant among many patients suffering from various types of cancers, which is why the number of patients treated by immunotherapy as a single therapy has dwindled [129]. To solve this problem, the role of miRNAs has become more prominent in the diagnosis, treatment, and mechanism of immune responses. Regulation of monocyte differentiation and maturation is a quintessential role for miRNAs in adaptive immunity. This immunity is important for a qualified response against cancer. miR-17-5p, miR-20a, and miR-106a are considered the main miRNAs on this issue owing to their ability to target the expression of the transcription factor (TF) [130]. Several TFs were detected to curb the expression of the miRNAs (miR-21, miR-155, miR-424, and miR-17-92), which pertain to the regulation of monocyte differentiation by controlling the MAPK, TGF-β, and JAK-STAT signaling pathway [131].

Macrophages are divided into two opposite types: M1 and M2. Macrophages of M1 type affect T-helper-mediated immune responses, which control tumor growth or increase tumor eradication. miR-146a and miR-21 target adaptor molecules in TLR/NF-κB pathway, which in turn leads to weakening M1 polarization. Moreover, miR-146a is involved in the downregulation of IL-1R-associated kinase 1 and TNF receptor-associated factor 6. On the other hand, M2 macrophages are responsible for the mediation of antiinflammatory and pro-tumorigenic responses, which are activated by IL-4 and IL-13. M2 macrophage polarization is derived by miR-155 by targeting the suppressor of cytokine signaling 1 (SOCS1). miRNAs also have pivotal roles in the activity of other immune cells, such as NK cells and checkpoint molecules (Table 4) [129].

**Table 4:** The function of miRNAs in different immune cells [129]

Function	miRNAs
Inhibition of monocyte differentiation of maturation	miR-17-5p, miR-20a, miR-106a
Activation of monocyte differentiation	miR-155, miR-21, miR-17-92, miR-424
M1 polarization	miR-21, miR-146a
M2 polarization	miR-155, miR-125a/b
Maturation and regulation function of NK	miR-155
Regulation of NK from CD34 +	miR-181
Cytotoxicity of NK cells	miR-30e, miR-378, miR-27a-5p
Regulation of the expression of PD-L1	miR-34a-5p, miR-138-5p, miR-200, miR-424, miR-513
Regulation of the expression of PD-1	miR-135-5p
Regulation of CTLA-4	miR-138-5p

## Combining therapeutic methods in breast cancer cases

Even though single-agent therapies have shown promising results in cell lines and preclinical models, their use in aggressive TNBC has been hindered by extensive heterogeneity, which leads to drug resistance. Since combined drug therapy (CDT) has been proven to be helpful in clinical trials owing to enhanced pathological clinical response (PCR), progression-free survival (PFS), and overall survival (OS) in different cancers, it has become relatively popular as a therapeutic approach [118]. Such novel methods would bring about various advantages over conventional anticancer treatments. These approaches will be discussed in detail in the following sections.

### Combination of immunotherapy with epigenetics to treat breast cancer

As a novel strategy in CDT, a combination of immunotherapy with epidrugs has been considered the most effective among breast cancer cases [93]. To understand why CDT has been deemed a high-efficacy method, immunotherapy and epidrugs should be assessed separately. One of the drawbacks of immunotherapy is that many patients (over 40%) are unresponsive to the treatment. The second disadvantage is the relapse of the disease in treated patients. For instance, mutations in the JAK/STAT pathway may occur in some cases and end with resistance to the treatment. Likewise, using epidrugs can have side effects such as lymphopenia [119].

Manuela et al. found that combining HDAC inhibitors with PD-1 and CTLA-4 blockade agents could increase TIL infiltrations, tumor apoptosis, and tumor regression [132]. According to available evidence, the combination of epigenetic drugs (such as HDACis and DNMTis) and immune therapeutics is beneficial, as DNMTi and HDACi modulate the immune response and can prevent acquired resistance to immunotherapy. Given these circumstances, they can act synergistically on both cancer cells and immune cells to ameliorate antitumor responses [120].

It is noteworthy that, in some 4T1 breast cancer cases, the disease could not be treated solely by using anti-PD-L1 or anti-CTLA-4 agents. However, the combination of these therapeutics with HDACi entinostat has been shown to be profoundly effective for the treatment of breast cancer. This synergistic effect has led to the evaluation of a combination therapy, which includes the atezolizumab anti-PD-L1 antibody and HDACi entinostat among patients suffering from TNBC in phase-1 and phase-2 clinical trials. The other combinatorial strategy is the co-administration of anti-CTLA-4 with HDACi to treat breast cancer. It has recently been identified that HDAC inhibitors can induce antibody responses targeting CTLA-4. These antibodies could upregulate the recognition and obliteration of tumor cells. In addition, CTLA-4 is able to bind to CD80 and CD86, which are both expressed on the surface of tumor cells and APCs. It was also suggested that HDAC upregulates CD80 and CD86, which induces enhanced immunogenicity of tumor cells and obliteration of tumor cells. These properties make them a practical strategy to target CTLA-4 and treat breast cancer. Combining ipilimumab with entinostat for breast cancer treatment is the epitome of combining epidrugs with immunotherapy. It should be underlined that HMTis and HDMis (target EZH2) are other epidrugs that can be combined with immunotherapy to abolish the limitations of immunotherapy. For instance, atezolizumab (targets the PD-L1) is combined with tazemetostat (inhibits the EZH2) in the treatment processes [121]. Goswami et al. have revealed the other benefit of combining the inhibitors of EZH2 with immunotherapy agents. They found that combination therapy could reduce the number of immunosuppressive cells and promote the therapeutic efficacy of antibodies that target CTLA-4 [122] (Table 5).

**Table 5:** New treatment strategies in breast cancer

New/future treatment strategies			
	Therapy	Mechanisms of action	References
Biological agents	DNA methyltransferases (DNMTi) (e.g., azacitidine and decitabine)	Re-expression of MHC molecules, tumor antigens, and changing cytokine production Re-expression of endogenous ERα and PRs Reversing MHC1 gene promoter methylation	[97, 119]
	Histone de-acetylation inhibitors (HDACi) ( e.g., vorinostat, romidepsin, panobinostat, and belinostat)	Increasing the expression of MCH molecules on tumor cells Activation of the process of regulatory T cells Increasing NK cell activity Trapping cell cycle in G1 and G2 phase	[93, 99, 119]
	LSD1 inhibitors (e.g., TCP, ORY-1001, GSK-2879552, IMG-7289, INCB059872, CC-90011, and ORY-2001)	Reprogramming tumor-associated macrophages into M1	[100, 123]
	Histone methyltransferase inhibitors (HMTis)	Suppressing EZH2 Increasing MDSCs in the tumor microenvironment	[101]
Synthetic agents	BET inhibitors (e.g., I BET 151(GSK1210151A), I-BET 762 (GSK525762), OTX-015, TEN-010, CPI-203, and CPI-0610.)	Diminishing NF-κB activity, cytokine generation, and PD-L1 expression Suppressing BATF	[102, 133]
Cell therapy	Estrogen receptor modulators (e.g., tamoxifen and fulvestrant) and aromatase inhibitors (e.g., anastrozole, letrozole, and exemestane)	Trapping cell cycle in G0 and G1 phase Competing with 17β-estradiol (E2) at the receptor site Blocking the enzyme aromatase, thereby reducing the levels of E2, E1, and E1S both in the periphery and in the mammary tissue	([130], [125]
	Monoclonal antibodies (e.g., trastuzumab and pertuzumab)	Inhibiting HER1-HER2 dimerization Enhancing ADCC reaction	[93]
Combinational therapy	Tyrosine kinase inhibitors (TKIs) (e.g., lapatinib and neratinib)	Reversibly binding to the cytoplasmic ATP-binding sites of EGFR/HER1 and HER2 receptors, thereby blocking tyrosine kinase phosphorylation	[126]
Epigenetic therapy	Immunotherapy (e.g., atezolizumab and pembrolizumab)	Agglutination of tumor cells, leading to phagocytosis Immobilization of tumor cells culminate in inhibiting tumor invasion and spreading Phagocytosis, stemming from binding to Fc receptor on macrophages, so-called opsonization Cytotoxicity via NK cells destroying tumor cells Tumor lysis phagocytosis by activation of the complement system Neutralization of active substances	[127, 128]

## Conclusion

Despite the improved knowledge on the biology of breast cancer, treating patients suffering from breast cancer has remained a challenge. Breast cancer is also prevalent among women who have no family history of disorders, including inherited BRCA1/2 mutations. This observation drew attention to how epigenetic processes like DNA methylation, histone modification, and even microRNAs (miRNAs) can affect breast cancer pathophysiology, diagnosis, and treatment. However, single-agent therapy, which has had a positive impact on cell lines and preclinical models, has not been approved for aggressive TNBC. One of the snags ahead for immunotherapy is that many patients do not response to the treatment (over 40%). This is mainly due to the heterogeneity of the tumor cells, which could lead to drug resistance. CDT has become popular owing to the reported improvement in PCR, PFS, and OS in different cancers. In this line, combination of immunotherapy and epidrugs is a novel strategy in the treatment of CDT breast cancer. Thus, gaining a better understanding of the genetic, epigenetic, and biological processes contributing to breast cancer pathogenesis is imperative to identify more reliable diagnostic and prognostic biomarkers to treat metastatic and recurrent breast cancers.

## Data Availability

The datasets used and/or analyzed during the current study are available from the corresponding author on reasonable request.

## Abbreviations

miRNAs: MicroRNAs
ER: Estrogen receptor
PR: Progesterone receptor
GPER1: G-protein-coupled estrogen receptor 1
TKIs: Tyrosine kinase inhibitors
MAPK: Mitogen-activated protein kinase
PI3K: Phosphatidylinositol-3-kinase
GSK: Glycogen synthase kinase
FRP1: Frizzled-related protein 1
DKK1: Dickkopf 1
DNMT: DNA methyltransferase
Eph: Ephrin
JAK: Janus kinase
STAT: Signal transducer and activator of transcription
VEGF: Vascular endothelial growth factor
MMP: Matrix metalloproteinase
NF-κB: Nuclear factor kappa-light-chain enhancer of activated B cells
HIF-1α: Hypoxia-inducible factor 1-alpha
EMT: Epithelial–mesenchymal transition
MHC: Major histocompatibility complex
HDACis: Histone deacetylases
STING: Stimulators of interferon gene
BET: Bromodomain and extraterminal
CTLA-4: Cytotoxic T-lymphocyte-associated protein 4
PD-1: Programmed cell death protein 1
PD-L1: Programmed death ligand 1
NK: Natural killer
NKG2D: Natural killer group 2D
TNBC: Triple-negative breast cancer
CSCs: Cancer stem-like cells
TRAIL: TNF-related apoptosis-inducing ligand
PTEN: Phosphatase expression and tensin homolog on chromosome 10
TGF-β: Transforming growth factor-beta
SOCS1: Suppressor of cytokine signaling 1
CDT: Combined drug therapy
